# The mediating effects of motivation on the relations between occupational stress and physical activity among underresourced afterschool program staff

**DOI:** 10.1186/s12889-024-17800-x

**Published:** 2024-01-30

**Authors:** Anqi Deng, Nicole Zarrett, Allison M. Sweeney

**Affiliations:** 1 Behavioral Medicine Research Group, Department of Psychology, College of Arts and Sciences, University of South Carolina, 1330 Lady Street, Suite 400, 29201 Columbia, SC USA; 2 Department of Psychology, University of South Carolina, Columbia, USA; 3 Department of Biobehavioral and Nursing Science, College of Nursing, University of South Carolina, Columbia, USA

**Keywords:** Self-determination motivations, Moderate-to-vigorous physical activity, Adults, Afterschool program, Mediating effect

## Abstract

**Objective:**

Guided by Self-Determination Theory, this study aimed to examine the potential mediating effects of autonomous and controlled motivations on physical activity (PA) experiences of afterschool program (ASP) staff with occupational stress.

**Method:**

A total of 58 ASP staff provided full data. Staff occupational stress and self-determination motivations for PA were assessed. Participants’ daily moderate-to-vigorous physical activity (MVPA) was measured using accelerometer wear. A path analysis was used to address the research purpose.

**Results:**

Occupational stress negatively and indirectly predicted daily MVPA which was mediated by controlled motivation (*β* = − 4.15, *p* <.05). Autonomous motivation directly and positively predicted daily MVPA across all types and levels of ASP staff occupational stress (*β* = 9.93, *p* =.01).

**Conclusions:**

Autonomous motivation is a powerful predictor of staff PA levels despite the degree to which they experience stress. In contrast, controlled motivations are more vulnerable to occupational stress, and can lead to lower MVPA.

**Trial registration:**

Connect Through PLAY: A Staff-based Physical Activity Intervention for Middle School Youth (Connect). https://clinicaltrials.gov/ct2/show/NCT03732144. Registered 11/06/2018. Registration number: NCT03732144.

## Introduction

Within the United States, about 42.2% of adults have obesity. This is the first time in recorded history that the national rate has passed the 40% mark [1]. Physical activity (PA) is one of the most effective ways of reducing obesity rates, sustaining good health, and preventing disease and mental illness [2, 3]. Psychological stress has been identified as a primary barrier to adult health behaviors, predicting less PA and more sedentary behavior (76.4%) [4]. In particular, a growing literature has indicated that elementary and secondary school teachers experience significant levels of occupational stress [5] and high teacher occupational stress is related to burn-out rate [6], low levels of PA [7], and mental health issues [7]. Program staff of after school programs (ASPs) are charged with many of the same responsibilities and challenges that teachers face during the school day and thus, are likely to experience several of the same, and potentially, additional types of occupational stressors, especially within underresourced schools. However, no research to date has examined ASP staff experiences of occupational stress or the impacts of occupational stress on ASP staff PA. ASP staff serve as important role models and key facilitators of youth health behaviors [8, 9] with high quality afterschool programming shown to facilitate improvements in youth PA, social affiliation, and social emotional skills [10, 11]. Therefore, examining factors that can mediate the impact of stress on PA of ASP staff is not only critical for supporting staff physical and mental health but can also be beneficial to the children and youth who they work with.

ASPs are commissioned to promote mental and physical health for children and adolescents [12] and have substantial access to the over 7.8 million children and adolescents in the United States who are enrolled in ASPs [13]. However, ASPs within underserved communities face great challenges such as insufficient material or human resources, high rates of staff burnout, lack of continuing professional development trainings, and consequently, high rates of general stress for ASP staff [9]. As a key health issue, staff stress, in turn, can impair staff daily PA engagement and interfere with adoption and implementation of effective intervention to promote adolescents’ PA [12, 14]. Research evidence has documented that individual’s experience of stress can be a significant impediment to achieving healthful levels of PA [4, 12]. For example, a literature review of 55 studies indicated that the majority of studies (79.8%) found an inverse association of general stress (involving affective, physiological, biochemical, and cognitive-behavioral responses) and PA behavior among adults across the lifespan [4]. Given the negative association between high stress and PA, identifying factors that can mediate the negative impact of stress on PA has both theoretical and clinical significance. Self-determination theory (SDT) is a framework that has been applied to understand intrapersonal and motivational factors as potential mediators of stress on other outcomes including the stress-illness relationship [15–17], stress-mental health relationship [18], and negative school learning contexts on students’ behavioral, affective, and cognitive outcomes [19]. However, no studies to date have examined the mediating effects of self-determination motivations on the relations between stress and daily PA.

### Stress in afterschool program staff

Psychological stress is defined as a set of emotions that include feelings of threat, challenge, or harm that impacts a person’s biological, physical, or psychological well-being [20]. The high levels of occupational stress experienced by teachers is well-documented. Teachers in disadvantaged communities (high-crime neighborhoods, low social economic status, minority status) are at the highest risk of stress and report feeling over-burdened and under-prepared for the multiple roles they must fulfill inside and outside of the primary task of teaching [21, 22]. Although understudied, ASP staff likely experience similar occupational stressors as teachers, serving as front line socializers responsible for implementing curriculum, meeting the academic and social-psychological needs of youth, managing behavior, and interfacing with parents, teachers, and other school administrators.

There are three types of factors that are proposed as primary contributors to teachers’ and program staffs’ occupational stress levels: (a) work-related stress; (b) time management; and (c) discipline and motivation [23]. Work-related stress occurs when the work demands offered do not match teachers’ knowledge, skills, or abilities [23]. Time management entails having to balance the various aspects of one’s position to assess needs, set goals, manage the work schedule, and prioritize and plan tasks to achieve goals [23]. Teachers who experience time management stress are those who feel overcommitted, feel pressure during multi-tasking, and have little time for work-life balance [23]. Discipline- and motivation-related stress involves two aspects of teacher-student interaction. The first aspect is discipline which involves the demands on teachers to manage discipline issues in the classroom, monitor student behavior, and deal with poorly defined discipline policies in their schools [23]. The second aspect is related to student motivation issues where teachers may experience particularly high levels of stress when instructing students who are unmotivated or poorly motivated [23]. These three types of stress are strongly linked with teachers’ job performance [23, 24], however, few studies have examined the impact of this occupational stress on teacher or program staff PA nor has the potential role of program staff’s motivation factors as a mediator against the deleterious effects of stress on PA been considered.

### The mediating effect of motivation for physical activity

SDT [15], a framework for understanding human motivation by focusing on the importance of human inner resources for development and behavioral regulation, has been widely applied for understanding individuals’ PA behaviors [25]. Deci and Ryan (2000) propose four types of motivation that form a continuum ranging from the most “controlled” extrinsically-driven motivation to the most “autonomous” (i.e., self-determined) intrinsically-driven motivation. Specifically, motivation is autonomous when it is engaged in for enjoyment or fun (intrinsic motivation), when it is integrated into behavior and an individual’s self-identity (integrated regulation), or when it is accepted by the individual as the value of the behavior (identified regulation). In contrast, two non-self-determined types of extrinsic motivation, that is external motivation (i.e., acting to satisfy an external contingency) and introjected motivation (i.e., acting to avoid guilt or shame), have been identified as controlled motivation [15, 26, 27]. Scores representing either overall relative autonomy or summary scores for autonomous and controlled motives are often applied to examining the distinctive characteristics of each motivation type [27]. Autonomous motivation and controlled motivation are not mutually exclusive; individuals can have both types of motivation simultaneously at either a different or similar strength when doing an activity. Although autonomous motivation is more likely to lead to adaptive outcomes (e.g., enjoyment, engagement, and persistence) than controlled motivation, controlled motivations are still perceived as more desirable and effective than amotivation (i.e., an absence of intrinsic or extrinsic motivation) where individuals completely lack any volition to engage in an activity [26, 27].

Individuals with high autonomous motivation are likely to become effective in internalizing and integrating the regulation of an activity or activities [28]. Thus, considerable research has demonstrated positive relations between autonomous motivation and PA behavior and behavior change [29]. For example, AM Sweeney, DK Wilson, ML Van Horn, N Zarrett, K Resnicow, A Brown, M Quattlebaum and B Gadson [30] found an autonomous motivation focused approach targeting social connectedness, enjoyment of PA and positive intragroup competition is a promising approach for promoting PA among American African women (50.72 ± 13.66 years; 86.8% with obesity). Fenton et al. (2021) found that SDT based psychological intervention comprising autonomy-supportive strategies significantly increased engagement in MVPA among adult rheumatoid arthritis patients after three-month PA program. Lucas et al. (2021) found that SDT variables capturing the self-regulation of motivation and PA-related enjoyment were associated with PA behavior improvement among parents and their adolescent children. Another set of research studies found strong positive correlations between more autonomous types of motivation and the amount of walking and moderate-to-vigorous (MV)PA among adults, including those with physical or mental health conditions [31–34]. Additionally, inverse relations were observed between autonomous motivation and maladaptive outcomes, such as stress, burnout, depression, and loneliness among diverse adult samples (these studies included samples that were predominantly disadvantaged adult women; older adults (> 50 years), and cohorts with both men and women [4, 35, 36]. However, previous findings among studies that examined relations between controlled motivation and positive PA behavior change among adults are mixed. Although a set of studies found that controlled motivation had only small or no effects on positive PA behavior change [37, 38], another set of studies found that high levels of controlled motivation had significant positive impact on short-term positive PA behavior [39, 40]. In summary, the relations between controlled motivation and PA behavior are understudied, especially among ASP staff who work in underresourced high-stress communities.

### Purpose of current study

In particular, and important to this study, is the potential mediating effects of autonomous motivation and controlled motivations on the PA experiences of individuals’ with stress [4, 35, 36]. To fill this gap, this study aimed to examine the relations between three types of occupational stress, self-determination motivations, and daily PA among ASP staff who work within underresourced communities. Specifically, this study used cross-sectional data to investigate (a) whether occupational stress had a negative effect on staff daily PA behaviors; (b) whether high levels of autonomous and controlled motivations had positive effects on daily PA; and (c) whether autonomous and controlled motivations function to mediate the negative impact of stress on daily PA behaviors among ASP staff. It is hypothesized that autonomous motivation will positively and directly contribute to ASP staff daily MVPA and will mediate the negative effects of stress on daily MVPA. Additionally, it is hypothesized that controlled motivation will also directly contribute to MVPA but is expected to be more vulnerable to the deleterious effects of occupational stress.

## Methods

This study uses a cross-sectional study design to examine baseline ASP staff accelerometry-assessed daily PA and survey data that were collected as part of an ongoing large-scale five year randomized controlled clinical trial implemented within ASPs (see more details about intervention at Authors masked). During Y1 and Y2 (August 2018– March 2020), a total of 10 school- and community-based ASPs were randomly sampled with stratification criteria at site level from after school programs in the Southeastern United States. Stratification criteria specified that sites have to be mid-sized ASPs (enrollment between 30 and 60 youth) and youth enrollment of at least 50% of youth from underserved communities which is defined by free/reduced lunch and minority status.

### Participants

A total of 75 ASP staff were recruited from widely accessible national and state-based youth programming organizations (e.g., Boys and Girls Club) at the beginning of the school year. There were 99% of program staff within the 10 ASPs who met inclusion criteria and consented to participate. Staff attrition was due to staff leaving their jobs or being fired (10 staff), any new hired staff (to replace staff who left) were enrolled in our study (2 new staff participants). The University Institution of Research Board approved the study. Study assessments were conducted by a trained research team during the ASP hours (3pm– 6pm). On completion of the baseline data collection, nine staff members were excluded for either not meeting the accelerometer wearing time criteria (over 480 min of recorded data per day for at least 3 days) or for not completing the survey. Chi-Square tests indicated that there were no significant differences by gender [χ^2^ (1, 67) = 0.01, *p* =.90], race [χ^2^ (2, 67) = 1.39, *p* =.93], and age [χ^2^ (38, 67) = 37.48, *p* =.49] between those who were included in the data analysis and those were not included. The final sample was comprised of 58 staff (46.55% of male; average age = 34.12), who predominantly identified as European American (72.4%) or African American/Black (25.86%), with a small proportion of the sample identifying as multiracial, Asian, Pacific Islander or other (1.74%) and ranged from 16 to 53 years old (Mean = 26.53 years, SD = 10.62 years). All ASP staff had regular interaction with youth enrolled in the ASP and had consistent roles in implementing all components of the daily program curriculum.

### Data collection and procedures

The Connect measurement team (a team of undergraduate, post-baccalaureate and graduate student research assistants), under the supervision of the Connect Measurement Coordinator (Full time employee) collected baseline accelerometer and survey data in the first Fall term (September-October) of each school year. Accelerometers were administered to ASP Staff to wear for seven consecutive days on their non-dominant wrist while awake. Self-report surveys were administered using a basic paper-and-pencil format. A passive consent procedure was conducted in which staff were asked to sign a form only if they wish to opt out of wearing the accelerometer or fill out the survey. Staff could opt out of participation in the study at any time.

### Measures

#### Staff stress

Staff perceived stress levels were assessed using the three sub-scales (21 items) of the Teacher Stress Inventory (TSI; [41]. Researchers made only slight modifications to the original questions to make it applicable for ASP staff instead of teachers (e.g., “My afterschool program” replaced “My classroom”). The TSI includes three sub-scales: Time Management stressors are measured using eight items (α = 0.65; e.g., I easily over-commit myself); Work-Related Stressors are measured with six items (α = 0.77; e.g., My afterschool program is too big/demanding), and Discipline and Motivation stressors are measured with six items (α = 0.77; e.g., I feel frustrated because of discipline problems in the program). Each item is scored using a 5-point scale ranging from 1 (no impact, not noticeable) to 5 (major impact, extremely noticeable). Items on each subscale were summed and averaged to create a total subscale score with higher scores indicative of greater amounts of perceived stress. Internal consistency reliability was acceptable in the current study (α > 0.60).

#### Daily physical activity

Program staff participants’ daily MVPA was measured using the ActiGraph GT3X (ActiGraph LLC) accelerometer. Staff were instructed by researchers to wear the accelerometer for seven consecutive days on their non-dominant wrist while awake. Only staff that met the wear criteria for valid data, defined as wearing the accelerometer for at least 8 h per day for at least three days, were included in the analyses [42]. The accelerometry data were downloaded using ActiLife software (Version 6.13.3; ActiGraph LLC) and served as a raw data format containing acceleration data. The raw data were then read and summarized in R software using the GGIR package with the Euclidean Norm Minus One metric [43]. Criteria developed by M Hildebrand, VH VT, BH Hansen and U Ekelund [44] were used to determine PA intensity: 201 to 707 mg for moderate PA, and ≥ 707 mg for vigorous PA.

#### Motivation toward physical activity

Autonomous motivation and controlled motivation for PA was measured using the Behavioral Regulation in Exercise Questionnaire (BREQ; [45] self-report measure. BREQ includes four subscales with 15 items measuring four motivational regulations which include intrinsic motivation (four items; e.g., I exercise because it’s fun), identified regulation (four items; e.g., It’s important to me to exercise regularly), introjected regulation (three items; I feel guilty when I don’t exercise), and external regulation (four items; I feel under pressure from my friends/family to exercise). Each item is scored using a 6-point scale ranging from 1 (strongly disagree) to 6 (strongly agree). Items for each subscale were first summed and averaged to create a total subscale score. Then the subscale scores for intrinsic motivation and identified regulation were summed and averaged to create a mean score for autonomous motivation and the subscale scores for introjected regulation and external regulation were summed and average to create a mean score for controlled motivation. The internal consistency reliability for autonomous motivation (α = 0.84) and controlled motivation (α = 0.76) were acceptable in the current study.

### Data analyses

**Fig. 1:**
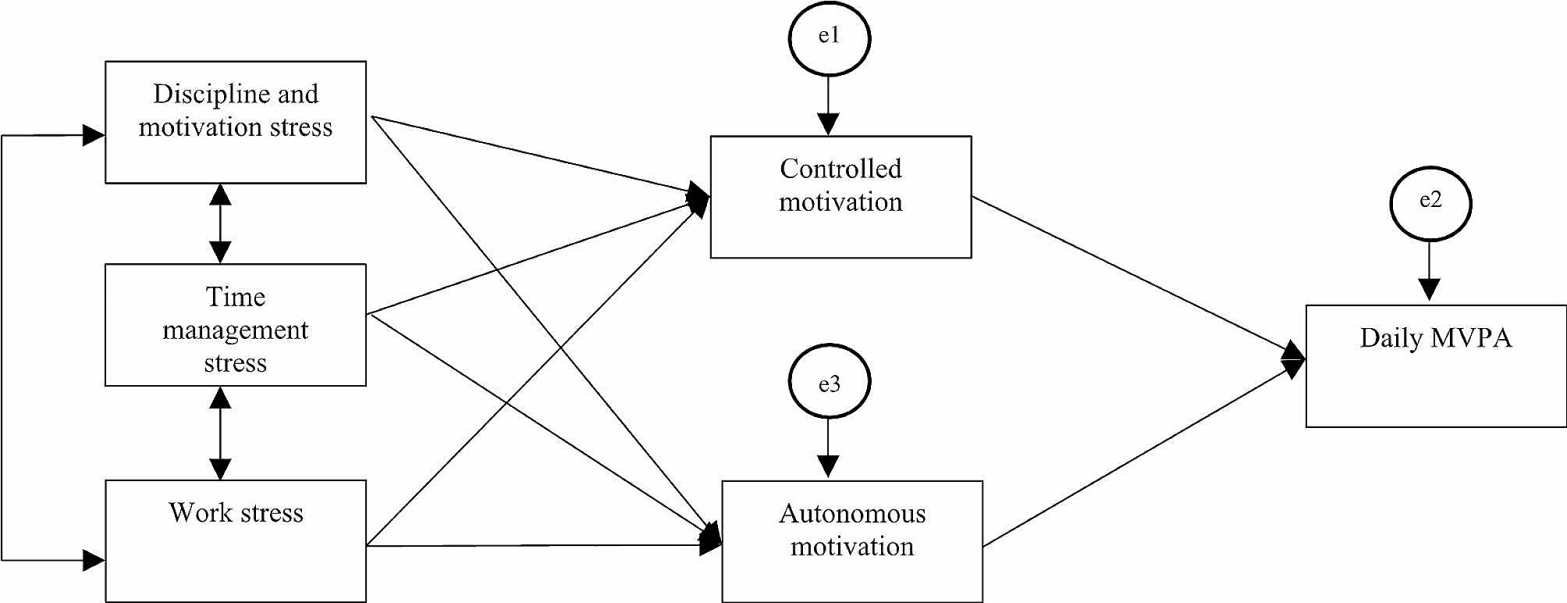
Path analysis model

Descriptive statistics analysis was used to explore the variables. A path analysis was used to simultaneously analyze the relations between the three types of ASP staff occupational stress, and the direct and indirect effects of staff stress on daily MVPA, as mediated by staff PA controlled or autonomous motivational orientations. Figure 1 presents the hypothetical path analysis model. It is most appropriate to conduct a path analysis (rather than use structural equation modeling) because all the variables in the model are observed characteristics or behaviors. Kline (2011) states that an adequate sample size should be 10 times the amount of the parameters in a path analysis [46]. Thus, given that the analyses included five parameters, a sample size of 54 provides adequate statistical power for the present analyses. The following indices and standards were used for a proper model fit, Chi-square (*p* >.05), Root Mean Square Error of Approximation (RMSEA; < 0.05), Comparative Fit Index (CFI > 0.90), and Tucker-Lewis index (TLI; > 0.90) [46]. Pearson correlation analysis was used to examine the relations among all variables. Linear regression analysis was employed to examine the direct effects of stress on daily MVPA. The path analysis was conducted using IBM SPSS Amos 27, the Pearson correlation analysis and linear regression analysis were conducted using IBM SPSS 27(IBM SPSS Inc., Chicago, IL, 2020).

## Results

### Descriptive statistics and bivariate correlations

On average, ASP staff spent 73.63 min per day in MVPA. Table 1 presents the descriptive statistics of all variables. The skewness indices (ranged between − 2 and 2) and the kurtosis indices (ranged between − 7 and 7) indicated no violation of the univariate normality assumption [46]. There were 62.1% of staff who perceived at least some impact from work-related stress (3.4% reported great-to-major impact), 5.2% of staff who reported an impact of time management stress (0% reported great-to-major impact), and 63.8% of staff who reported at least some impact from discipline and motivation stress (5.2% reported great-to-major impact). Table 2 presents the correlations between the variables included in the model. Work-related stress was positively correlated with discipline and motivation stress, time management stress was positively correlated with autonomous motivation. Both controlled and autonomous motivations were positively related with daily MVPA. The linear regression analysis results showed that work stress (β = -3.08, *p* =.59), time management stress (β = -1.69, *p* =.74), and discipline and motivation stress (β = -2.73, *p* =.60) did not significantly predict daily MVPA.

**Table 1:** Descriptive results for all variables

	Mean	SD	Skewness	Kurtosis
Autonomous motivation	3.70	0.98	− 0.59	0.31
Controlled motivation	2.89	0.84	− 0.76	0.04
Discipline and motivation stress	2.14	1.15	0.78	− 0.09
Time management stress	2.42	1.12	0.33	− 0.60
Work-related stress	2.04	1.04	0.73	− 0.15
Daily MVPA	73.63	32.94	0.78	− 0.09

**Table 2:** Correlations between all variables

Variables	1	2	3	4	5	6
1 - Work-related stress	-					
2 - Time management stress	0.13	-				
3 - Discipline and motivation stress	0.67*	0.14	-			
4 - Controlled motivation	0.14	− 0.13	− 0.13	-		
5 - Autonomous motivation	0.06	0.33*	− 0.03	0.25	-	
6 - Daily MVPA	− 0.17	− 0.07	− 0.17	0.29*	0.35**	-

Note: * *p* <.05; **p <.01

#### Path analysis results

**Fig. 2:**
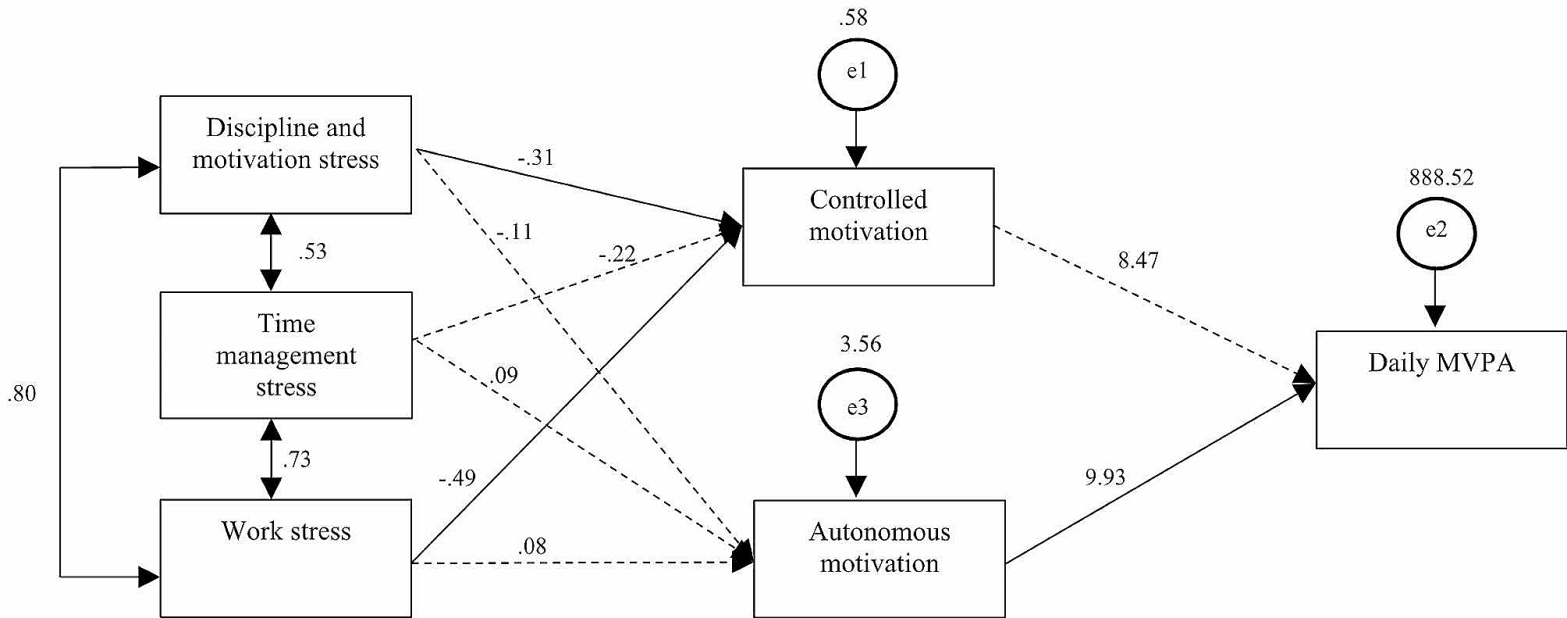
Path analysis model direct effect results

**Fig. 3:**
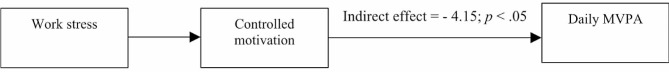
Path analysis model for significant indirect effect results

The initial model was shown to fit the data well: χ2 = 8.18, df = 4, *p* =.08; CFI = 0.95; RMSEA = 0.04; TLI = 0.95. As shown in Table 3; Figs. 2 and 3, the path analysis results revealed that discipline and motivation stress and work-related stress significantly and negatively predicted controlled motivation (*β* = − 0.31, *p* =.01; *β* = − 0.49, *p* =.002, respectively) but not autonomous motivation (*β* = 0.09, *p* =.46; *β* = 0.08, *p* =.69, respectively). Time management stress did not significantly predict either controlled motivation (β = − 0.22, *p* =.06) nor autonomous motivation (*β* =-0.02, *p* =.53). Autonomous motivation positively and significantly predicted daily MVPA (β = 9.93, *p* =.01) while controlled motivation did not (*β* = 8.47, *p* =.07). Results further showed that work stress negatively and indirectly predicted daily MVPA which was mediated by controlled motivation (*β* = − 4.15, *p* <.05). Autonomous motivation did not mediate any of the negative impact from occupational stress on daily MVPA.

**Table 3:** Standardized regression coefficients for the path analysis model

Estimator	Effects	*p*
**Autonomous motivation**		
Discipline and motivation stress	0.09	0.46
Time management stress	− 0.02	0.53
Work stress	0.08	0.69
**Controlled motivation**		
Discipline and motivation stress	− 0.31	0.01
Time management stress	− 0.22	0.06
Work stress	− 0.49	0.002
**Daily MVPA**		
Autonomous motivation	9.93	0.01
Controlled motivation	8.47	0.07

## Discussion

This study set out to examine the relations between occupational stress, self-determination motivations, and daily MVPA among program staff who are working in underresourced ASPs. Specifically, this study aimed to address several gaps in previous research by examining: (a) whether occupational stress had a negative effect on staff daily PA behaviors; (b) whether high levels of autonomous and controlled motivations had positive effects on daily PA; and (c) whether autonomous and controlled motivations function to mediate the negative impact of stress on daily PA behaviors among ASP staff. The results aligned with our hypotheses and showed that occupational stress did not have direct effects on daily MVPA, but operated through its inverse effect on controlled motivation orientations. In particular, work stress and discipline and motivation stress had significant effects on staffs’ controlled motivation, with higher levels of stress linked to lower levels of controlled motivation for PA. Consequently, work stress (e.g., my afterschool program is too big/demanding) was found to indirectly predict less daily MVPA through the negative impact it has on controlled motivation. These findings are clinically relevant as the ASP staff in our study reported similar cumulative occupational stress levels as other high-stress populations including first-year teachers [47] and early childhood education teachers [48, 49] and work-related stressors were identified as a particular impactful experience for the majority of staff (62% of staff) as compared to the other occupational stressors measured. In contrast, time management stress (e.g., I have little time to relax/enjoy the time of day) was not identified as an impactful stressor for the majority of staff in our sample and was not related to staffs’ self-determination motivations or MVPA.

Overall, findings suggest that controlled forms of SDT motivation are vulnerable to environmental/work stressors. In contrast, the three types of occupational stress were not predictive of the degree to which an adult had autonomous motivation for PA, and autonomous motivation was found to have a direct positive impact on daily MVPA. The findings indicate that those who have higher autonomous motivation engage in more MVPA despite occupational stress they may experience. Findings support previous research indicating that autonomous motivation is a highly effective motivation orientation for promoting PA [29, 30] and contributes to our understanding of the resilience of autonomous motivation within the context of environmental/work stressors. Future studies are needed to examine whether autonomous forms of motivation for PA are resilient against other types of stressors or other barriers. The findings inform important mechanisms for PA interventions, especially for individuals who work in highly stressful and/or underresourced environments such as the ASP program staff in our study.

Physical activity is arguably the most promising non-pharmacological, noninvasive, and cost-effective method of health-promotion. The Physical Activity Guidelines for Americans outlined recommendations of at least 150–300 min/week of MVPA and two days of muscle strengthening activity for adults [50]. ASPs are argued to have the ability to reach underserved youth and adult populations that are at risk for low levels of daily PA [51]. Several studies have documented that staff PA behaviors can have a significant impact on the number/proportion of youth who are physically active during ASP hours, that is, youth are more engaged in MVPA when staff are also engaged [51, 52]. Therefore, understanding and maintaining ASP staff MVPA is not only meaningful for adult health promotion, but also for ASP curriculum designers and researchers in the service of promoting youth PA behavior change. In the current study, on average, staff were accumulating 73.63 min of daily MVPA which was aligned with the recommendation of 150–300 min/week. In addition, approximately 46.2% of ASP staff report average and above average autonomous motivation and 56.9% of ASP staff were accumulating average daily MVPA time above 60 min. The findings indicate that autonomous motivation has positive relationships with PA behavior, with higher levels of autonomous motivation linked to higher levels of daily MVPA.

A primary implication of this study’s findings is the provision of additional evidence that supports the importance of adult health promotion intervention and programming to focus on facilitating autonomous motivation orientations for PA. According to SDT-based PA promotion frameworks, autonomous motivation is perceived to be the origin or driver of the belief-based antecedents in PA behavior [53, 54]. Specifically, autonomous motivation toward a given behavior or activity tends to lead to approach-oriented beliefs about performing the behavior and the formation of intention to engage in the behavior in future. Hagger and colleagues (2016) further argue that the mechanisms that underpin the link between self-determined behavioral regulations and belief-based antecedents is derived from the processes of psychological needs satisfaction and internalization [54]. They propose that if an individual has previously experienced an activity as autonomously motivated and internalized it into the repertoire of behaviors that satisfy psychological need, they will be more likely to pursue future opportunities to engage in the activity as a means of continued need satisfaction regardless of environmental or social resources or barriers [54]. The results of the current study confirmed the theoretical assumptions and expanded the results of previous studies to a special population– underresourced afterschool program staff, suggesting that autonomous motivation is a positive predictor of PA and a key mechanism to be considered for health promotion, especially among adults within occupational settings with a significant number of stressors. The findings are meaningful for staff-health curriculum and teacher/staff continuing professional workshop design.

Findings align with previous studies that found controlled motivation did not significantly and directly impact daily MVPA [55, 56]. The current study findings also suggest that controlled motivation orientations for PA are more vulnerable to external stressors, likely explaining, at least in part, the mixed findings associated with controlled motivation and PA. Unlike the intrinsic nature of autonomous motivation, controlled motivation emanates from self-imposed pressures such as shame or pride, or from external pressures and controls to engage in an activity [57]. Given individuals with high control-motivational orientation are sensitive to environmental or personalized factors, it is not surprising that our findings indicate that their PA behavior is more vulnerable to occupational stressors [58]. In particular, work stressors (e.g., feeling overwhelmed by work-related demands) and stressors associated with managing youth disciplinary and motivation issues directly impede/impair the controlled, or extrinsic, aspects of staff PA motivation. However, stressors related to Time Management, which are more self-driven types of stressors (e.g., over-committing oneself at work) did not impact motivation. These findings may reflect previous research which has found that unpredictable stressors that reside outside one’s personal control (as compared to stressors residing within one’s locus of control) can lead to greater personal experiences of stress and burnout [59, 60]. Specifically, occupational stressors, namely work stress, may reduce the number or deplete the impact of extrinsic motivators on PA engagement. Therefore, the PA of ASP staff/teachers who have high controlled-motivation orientations towards PA are particularly impaired by occupational stress, especially when work demands exceed one’s capacity. Taken together, the results indicate that attention towards establishing a positive social-motivational climate that supports ASP staff autonomous motivation needs for PA can have clinically significant impact on their daily MVPA. Future staff comprehensive training and staff health initiative intervention are needed to enhance staff autonomous motivation in order to overcome stress-related barriers to PA. In turn, improvements in staff motivation and engagement in PA, can position ASP staff as positive supports and role models for promoting increased PA of youth enrolled in ASPs (Authors Masked).

### Limitations

There are several limitations in this study. First, this study has a relatively small sample size among ASP staff who work within underresourced ASPs, which restricts the generalizability. Staff from ten underresourced ASPs from the southeastern United States - and the use of accelerometers to measure MVPA are strengths, however, results may not be generalizable to other ASP staff who are working in higher socio-economic communities and/or other regions. Future studies are needed to recruit a greater number of ASP staff participants across the country. Second, staff stress, autonomous motivation and controlled motivation for PA were measured via self-administered survey. Common method bias can be a potential limitation. Third, the current study applied a cross-sectional research design impeding the ability to draw definitive conclusions concerning the directionality or causality among the variables examined. Future studies are needed to examine the hypothetical model over time as well as include other critical metrics of occupational stress including duration and frequency of the stressors. Autonomous and controlled motivations are not mutually exclusive but rather, individuals can have both types of motivation in varying degrees when engaged in an activity. Therefore, future studies that examine ASP staff motivational orientation profiles across the four self-determined orientations and its impact on motivation and PA behavior could also be beneficial to understanding how these motivations operate together to promote participation and engagement.

## Conclusions

This study highlights the positive relationship between staffs’ autonomous motivation and their daily PA levels. Our study shows that autonomous motivation is a powerful predictor of staff PA levels despite the degree to which they experience occupational stress within underresourced ASPs. In contrast, work stress and discipline and motivation stress had inverse relationships with controlled motivation, with higher levels of stress linked to lower levels of controlled motivation for PA. Controlled motivation orientations towards PA can be impaired by stressors experienced in the highly stressful ASP workplace. Hence, future interventions that improve participants’ autonomous motivations are needed. Likewise, although reducing occupational stress may not be possible, finding mechanisms to minimize the impact of stress on an individual’s PA motivation, in particular, preventing it from impairing controlled motivations, can make a significant difference on staff PA behavior and health. Providing resources and supports that not only increase PA but help reduce occupational stressors within the ASP setting will not only improve staff health, but, through staff positive attitudes, values and behaviors, is likely to facilitate improvements in adolescents’ motivation and PA behavior during the afterschool hours. Future studies are also needed to apply classification system of motivational and behavior change techniques to design and assess the future ASP staff health interventions [61].

## Data Availability

A copy of survey instrument questions will be provided upon request to the corresponding author. Participant data are not publicly available due to human subject research protections but qualified academic researchers may send data requests to the corresponding author for review. All use of data would be subject to confidentiality and data-use agreements.

## Abbreviations

ASP: Afterschool program
MVPA: Moderate-to-vigorous physical activity
PA: Physical activity
SDT: Self-determination theory
TSI: Teacher Stress Inventory
BREQ: Behavioral Regulation in Exercise Questionnaire
RMSEA: Root Mean Square Error of Approximation
CFI: Comparative Fit Index
TLI: Tucker-Lewis index
